# Supplementary material for: Bayesian estimation of gene constraint from an evolutionary model with gene features
Source: bioRxiv. 2023 May 21:2023.05.19.541520. Preprint. [Version 1] doi: 10.1101/2023.05.19.541520 (PMC10245655; doi:10.1101/2023.05.19.541520)
Supplement: Supplementary file 1 [file NIHPP2023.05.19.541520v1-supplement-4.pdf]

bioRxiv preprint doi: https://doi.org/10.1101/2023.05.19.541520; this version posted May 21, 2023. The copyright holder for this preprint (which was not certified by peer review) is the author/funder, who has granted bioRxiv a license to display the preprint in perpetuity. It is made available under aCC-BY 4.0 International license.

Supplementary Material

A Empirical Bayes with `NGBoost`

Empirical Bayes overview

In the simplest version of empirical Bayes, we specify the form of the prior distribution and assume that that prior is shared across all genes—for example, for gene i we might assume the prior distribution is $s_{\text{het}}^{(i)} \sim \text{LogitNormal}(\mu, \sigma)$ with density $p_{\mu,\sigma}(s_{\text{het}}^{(i)})$. Where the $\text{LogitNormal}(\mu, \sigma)$ distribution is defined such that $\text{logit}(s_{\text{het}}^{(i)}) = \log(s_{\text{het}}^{(i)}/(1 - s_{\text{het}}^{(i)}))$ is normally distributed with mean μ and variance σ^2. We can then estimate μ and σ using the observed LOF data for each gene, $\boldsymbol{y}_1, \ldots, \boldsymbol{y}_M$, by maximizing the marginal likelihood:

$$\prod_{i=1}^{M} \int_0^1 p\left(\boldsymbol{y}_i \mid s_{\text{het}}^{(i)}\right) p_{\mu,\sigma}\left(s_{\text{het}}^{(i)}\right) \mathrm{d}s_{\text{het}}^{(i)}. \tag{1}$$

Next, we can compute the posterior distribution of $s_{\text{het}}^{(i)}$ for each gene,

$$p\left(s_{\text{het}}^{(i)} \mid \boldsymbol{y}_i\right) = \frac{p\left(\boldsymbol{y}_i \mid s_{\text{het}}^{(i)}\right) p_{\mu,\sigma}\left(s_{\text{het}}^{(i)}\right)}{\int_0^1 p\left(\boldsymbol{y}_i \mid s_{\text{het}}^{(i)}\right) p_{\mu,\sigma}\left(s_{\text{het}}^{(i)}\right) \mathrm{d}s_{\text{het}}^{(i)}}. \tag{2}$$

However, rather than learning the parameters for the prior from only the LOF data, we can also use gene features to learn gene-specific prior parameters, μ_i and σ_i. To do this, we used a machine learning approach, `NGBoost`, to learn functions f and g such that $\mu_i = f(\boldsymbol{x}_i)$ and $\sigma_i = g(\boldsymbol{x}_i)$, where $\boldsymbol{x}_i$ is a vector of gene features associated with gene i. In the next few sections, we will describe how we learned f and g.

`NGBoost`

`NGBoost` (Natural Gradient Boosting) is an approach for training gradient boosted trees to predict the parameters of a probability distribution [17]. Gradient boosted trees are a type of machine learning model typically used to predict outcomes, y, from features, X, producing point estimates such as predicting $\mathbb{E}[y \mid X]$; in contrast, `NGBoost` uses gradient boosted trees to predict $p(y \mid X = \boldsymbol{x})$ by learning parameters of $p(y \mid X = \boldsymbol{x})$ as functions of $\boldsymbol{x}$—in other words, `NGBoost` allows us to learn the full distribution of y conditioned on observing the features $\boldsymbol{x}$.

Specifically, for gene i, we assume the prior distribution is $s_{\text{het}}^{(i)} \sim \text{LogitNormal}(\mu_i, \sigma_i)$, with density $p_{\mu_i,\sigma_i}(s_{\text{het}}^{(i)})$. $\mu_i = f(\boldsymbol{x}_i)$ and $\sigma_i = g(\boldsymbol{x}_i)$ are functions of the vector of gene features $\boldsymbol{x}_i$ (f and g are parameterized as gradient-boosted trees). We chose this distribution as previous work has suggested that $s_{\text{het}}^{(i)}$ is distributed on a logarithmic scale [1,2,4], yet, $s_{\text{het}}^{(i)}$ is also bounded between 0 and 1. Both of these properties are enforced by the LogitNormal distribution. In Supplementary Note B, we develop a population genetic likelihood $p(\boldsymbol{y}_i \mid s_{\text{het}}^{(i)})$, where $\boldsymbol{y}_i$ is a vector that represents the observed frequencies of each possible loss of function variant for the gene. Then, with M genes

bioRxiv preprint doi: https://doi.org/10.1101/2023.05.19.541520; this version posted May 21, 2023. The copyright holder for this preprint (which was not certified by peer review) is the author/funder, who has granted bioRxiv a license to display the preprint in perpetuity. It is made available under aCC-BY 4.0 International license.

in the training set, the score that `NGBoost` maximizes during training is:

$$\sum_{i=1}^{M} S\left(\boldsymbol{y}_i; \mu_i, \sigma_i\right) = \sum_{i=1}^{M} \log p\left(\boldsymbol{y}_i\right) = \sum_{i=1}^{M} \log\left(\int_0^1 p\left(\boldsymbol{y}_i \mid s_{\text{het}}^{(i)}\right) p_{\mu_i,\sigma_i}\left(s_{\text{het}}^{(i)}\right) \mathrm{d}s_{\text{het}}^{(i)}\right). \tag{3}$$

To do this, `NGBoost` first initializes the parameters of f and g such that all genes have the same prior distribution. Next, `NGBoost` adopts a gradient descent approach to maximize the score function: for each iteration until training ends, `NGBoost` first computes the natural gradient of gene i's score with respect to the parameters μ_i and σ_i of $p_{\mu_i,\sigma_i}(s_{\text{het}}^{(i)})$, where the natural gradient of $S = S(\boldsymbol{y}_i; \mu_i, \sigma_i)$, is defined as:

$$\widetilde{\nabla} S \propto \mathcal{I}_{\mu_i,\sigma_i}^{-1} \nabla_{\mu_i,\sigma_i} S \tag{4}$$

where

$$\mathcal{I}_{\mu_i,\sigma_i} = \mathbb{E}_{s_{\text{het}}^{(i)} \sim p_{\mu_i,\sigma_i}}\left[\left(\nabla_{\mu_i,\sigma_i} \log p_{\mu_i,\sigma_i}\left(s_{\text{het}}^{(i)}\right)\right)\left(\nabla_{\mu_i,\sigma_i} \log p_{\mu_i,\sigma_i}\left(s_{\text{het}}^{(i)}\right)\right)^T\right] \tag{5}$$

is the Fisher Information Matrix for $p_{\mu_i,\sigma_i}(s_{\text{het}}^{(i)})$ and ∇_{μ_i,σ_i} represents differentiation with respect to μ_i and σ_i. Natural gradients take into account the underlying "information geometry" of the space of distributions in a way that standard gradients do not [84]. As an example, changing the variance of a Normal distribution from 0.1 to 0.2 is much more dramatic than changing the variance from 10.1 to 10.2. After computing the natural gradient, `NGBoost` fits a decision tree to each dimension of the natural gradient, updating μ_i and σ_i in the direction that most steeply increases the gene's score. While gradient-boosting algorithms (including `NGBoost`, by default) typically fit a single decision tree at each iteration, we allow `NGBoost` to fit one or more trees, which performs slightly better in practice (see "Training and Validation" in Methods).

Below, we summarize the training algorithm. Let $\mu_i^{(t)}, \sigma_i^{(t)}$ denote the parameters of the prior at training iteration t.

1. Initialize parameters for all genes, $i = 1, ..., M$:
   $\mu_i^{(0)}, \sigma_i^{(0)} = \text{argmax}_{\mu,\sigma} \sum_{i=1}^{M} S(\boldsymbol{y}_i; \mu, \sigma)$
2. For iterations $t = 1, ..., T$:
   (a) For each gene, calculate natural gradients of the score:
   $\widetilde{\nabla} S\left(\boldsymbol{y_i}; \mu_i^{(t)}, \sigma_i^{(t)}\right)$, whose two components we denote as $\widetilde{\nabla} S_\mu$ and $\widetilde{\nabla} S_\sigma$
   (b) Fit decision trees $f^{(t)}$ and $g^{(t)}$ on the natural gradients:
   $f^{(t)} = \text{fit}\left(\left\{\boldsymbol{x}_i, \widetilde{\nabla} S_{\mu_i}\right\}_{i=1}^{M}\right)$
   $g^{(t)} = \text{fit}\left(\left\{\boldsymbol{x}_i, \widetilde{\nabla} S_{\sigma_i}\right\}_{i=1}^{M}\right)$
   (c) Update the parameters for each gene, where η is a learning rate that is chosen by the user as a hyperparameter
   $\mu_i^{(t)} = \mu_i^{(t-1)} - \eta f^{(t)}(\boldsymbol{x}_i)$
   $\sigma_i^{(t)} = \sigma_i^{(t-1)} - \eta g^{(t)}(\boldsymbol{x}_i)$

bioRxiv preprint doi: https://doi.org/10.1101/2023.05.19.541520; this version posted May 21, 2023. The copyright holder for this preprint (which was not certified by peer review) is the author/funder, who has granted bioRxiv a license to display the preprint in perpetuity. It is made available under aCC-BY 4.0 International license.

Once training is complete, we obtain a learned prior with parameters $\mu_i^{(T)}, \sigma_i^{(T)}$, and can compute the posterior distribution of s_{het}

$$p\left(s_{\text{het}}^{(i)} \mid \boldsymbol{y}_i\right) = \frac{p\left(\boldsymbol{y}_i \mid s_{\text{het}}^{(i)}\right) p_{\mu_i^{(M)},\sigma_i^{(M)}}\left(s_{\text{het}}^{(i)}\right)}{p\left(\boldsymbol{y}_i\right)} \tag{6}$$

as well as the mean of this distribution

$$\mathbb{E}\left[s_{\text{het}}^{(i)} \mid \boldsymbol{y}_i\right] = \int_0^1 s_{\text{het}}^{(i)} p(s_{\text{het}}^{(i)} \mid \boldsymbol{y}_i) ds_{\text{het}}^{(i)} \tag{7}$$

To compute 95% Credible Intervals, we compute the CDF of the posterior distribution using Pytorch's `cumulative_trapezoid` function [85]. Then, the 95% Credible Interval per gene is defined as $[\text{lb}^{(i)}, \text{ub}^{(i)}]$ such that $P(s_{\text{het}}^{(i)} < \text{lb}^{(i)}) = 0.025$ and $P(s_{\text{het}}^{(i)} < \text{ub}^{(i)}) = 0.975$.

`NGBoost`— implementation details

To initialize parameters (step 1 in the training algorithm), we perform gradient descent with the AdamW optimizer [86] implemented in `PyTorch` [85] with the default settings and a learning rate of 5×10^{-4}. We initialize the optimization at $\mu = -5$ and $\sigma = 0.5$.

To compute the integrals in the score calculation, we use the `torchquad` package for numerical integration [87], which allows us to use `PyTorch`'s automatic differentiation system to compute gradients. We perform integration using Boole's rule, integrating from 5×10^{-8} to $1 - 5 \times 10^{-8}$ with 10^6 sample points.

The Fisher Information Matrix is approximated using a Monte Carlo approach: we sample s_{het} from the prior 1,000 times, compute the gradient for each sample, and approximate the expectation using the sample mean.

To flexibly fit decision trees at each training iteration, we use the `XGBoost` package, a library used for fitting standard gradient boosted trees [88]. In comparison to the default `NGBoost` learner, `XGBoost` supports missing features and allows for adjustment of numerous hyperparameters (see "Training and Validation" in Methods). In contrast to typical applications of `XGBoost`, we only allow a few (1-4) trees to be fit at each training iteration, as we are using `XGBoost` within a training loop rather than as a standalone approach for model fitting.

All distributions were implemented using `PyTorch`, and training was conducted with GPU support when available, with `tree_method = "gpu_hist"` for the `XGBoost` learners.

bioRxiv preprint doi: https://doi.org/10.1101/2023.05.19.541520; this version posted May 21, 2023. The copyright holder for this preprint (which was not certified by peer review) is the author/funder, who has granted bioRxiv a license to display the preprint in perpetuity. It is made available under aCC-BY 4.0 International license.

B Population Genetics Model

Overview of model

Some of the most commonly used measures of gene constraint (pLI [11], LOEUF [12]) are framed in terms of the number of unique LOFs observed in gene, O, relative to the number expected under a null model, E. While operationalizing constraint as some function of O and E captures the intuition that seeing fewer LOFs than expected is evidence that a gene is conserved, the numerical values of pLI and LOEUF are difficult to interpret. In practice this means that such measures can be useful for ranking which genes are important, but it makes it difficult to contextualize these results in terms of other types of variants, such as missense or noncoding variants, or copy number variants. Previous approaches have pioneered using a population genetics model in this context to obtain interpretable estimates, albeit with different technical details that we discuss below [1,2,4].

In order to obtain a more interpretable measure of constraint, we formalize constraint as the strength of natural selection acting against gene loss-of-function in a population genetics model. That is, we can ask how much fitness is reduced on average for an individual with one or two non-functional copies of a gene relative to individuals with two functional copies, following previous work [1,2,4]. To tie this concept of constraint to observed allele frequency data, we use a slightly simplified version of the discrete-time Wright Fisher model. This model contains mutation, selection, and genetic drift, and assumes that there are only two alleles and that the population is panmictic, monoecious, and has non-overlapping generations. While all of these assumptions are violated in humans (there are four nucleotides, population structure, two sexes, and overlapping generations), the model still provides a good approximation to allele frequency dynamics through time. If the allele frequency in generation k is f_k, then we model the allele frequency in the next generation via binomial sampling:

$$2N_{k+1}f_{k+1} \sim \text{Binomial}\left(2N_{k+1}, p\left(f_k\right)\right), \tag{8}$$

where N_{k+1} is the number of diploid individuals in generation $k+1$, with

$$p(f_k) := \frac{(1-s_{\text{het}})\widetilde{f}_k\left(1-\widetilde{f}_k\right) + (1-s_{\text{hom}})\widetilde{f}_k^{\,2}}{\left(1-\widetilde{f}_k\right)^2 + 2(1-s_{\text{het}})\widetilde{f}_k\left(1-\widetilde{f}_k\right) + (1-s_{\text{hom}})\widetilde{f}_k^{\,2}},$$

where $\widetilde{f}_k = f_k(1-\mu_{1\to 0}) + \mu_{0\to 1}(1-f_k)$ is the allele frequency after alleles change from non-LOF to LOF at rate $\mu_{0\to 1}$ and from LOF to non-LOF at rate $\mu_{1\to 0}$. The function $p(\cdot)$ arises from considering bidirectional mutation and approximating a model of diploid selection where the relative reproductive success of individuals with 0, 1, or 2 copies of the LOF are $1, 1-s_{\text{het}}$, and $1-s_{\text{hom}}$ respectively [13]. In practice, most LOF variants are extremely rare, and so it is exceedingly unlikely to find individuals homozygous for the LOF. This makes estimating s_{hom} as a separate parameter very difficult, and so we instead assume that $s_{\text{hom}} = \min\{2s_{\text{het}}, 1\}$. This is equivalent to assuming genic selection (i.e., additive fitness effects) with the constraint that an individual's relative fitness cannot be lower than 0.

Equation 8 fully specifies the model except for an initial condition. That is, we need to know what the distribution of frequencies is in generation 0. One mathematically appealing choice

bioRxiv preprint doi: https://doi.org/10.1101/2023.05.19.541520; this version posted May 21, 2023. The copyright holder for this preprint (which was not certified by peer review) is the author/funder, who has granted bioRxiv a license to display the preprint in perpetuity. It is made available under aCC-BY 4.0 International license.

would be to assume that the population is at equilibrium at time 0, but this seemingly straightforward choice results in nonsensical conclusions. To see why, if the mutation rates are low and selection is negligible, then at equilibrium, with extremely high probability the population will either be in a state where the frequency of the LOF allele is very close to zero or in a state where the frequency of the LOF allele is very close to one. If the mutation rates between the two alleles are close to equal, then these two cases happen roughly equally often. That is, we would expect there to be a $\sim$50% chance that the population is fixed or nearly fixed for the LOF mutation. If there are multiple independently evolving sites at which an LOF could arise (or if there are many more ways to mutate to an LOF state than a non-LOF state), then the chance that any of these sites is fixed or nearly fixed for an LOF rapidly approaches 100%. Under this equilibrium assumption, we thus reach the absurd conclusion that the mere act of observing a gene that is functional in a majority of the population is overwhelming evidence that the gene is strongly selected for. Another way of viewing this is that in reality we can only observe genes that are functional in an appreciable fraction of the population, and so we should somehow be conditioning on this event, whereas the equilibrium assumption looks at a given randomly chosen stretch of DNA and asks whether it could be a gene given some set of mutations. Indeed, any randomly chosen stretch of DNA could be made a gene through a series of mutations, but for any given stretch it would be extremely unlikely to be a functional gene, and the equilibrium assumption exactly captures how rare this would be.

We instead use the equilibrium of another process as the initial condition, which avoids these conceptual pitfalls. We assume the distribution of frequencies at generation 0 is the equilibrium conditioned on the LOF allele never reaching fixation in the population. We then compute the likelihood of observing a given present-day frequency while continuing to condition on non-fixation of the LOF allele. This assumption implies that no matter the current frequency of the LOF variant, we know that at some point in the past the population was fixed for the functional version of the gene, and the LOF variant can thus be thought of as being “derived” and the non-LOF variant “ancestral”. In the limit of infinitely low (but non-zero) mutation rates, this assumption become equivalent to the commonly assumed “infinite sites” model commonly used to compute frequency in population genetics [89]. In contrast to the infinite sites model, where the probability that any given site is segregating must be 0, our model allows us to compute the probability that a given site is segregating. Furthermore, we can easily model recurrent mutation which can be important for sites with large mutation rates (such as CpGs) and large sample sizes [90], whereas under the infinite sites model each mutation necessarily happens at a unique position in the genome, ruling out the possibility of recurrent mutation. Below we will write $p_{\text{DTWF}}(y \mid s_{\text{het}})$ for the probability mass function computed using this procedure, with “DTWF” representing Discrete-Time Wright-Fisher, and y being an observed LOF allele frequency.

Equation 8 is easy to describe and simulate under, and a very similar model has been used in an approximate Bayesian computation approach to estimate s_{het} [4]. While simulation is easy, computing likelihoods under this model is difficult for large sample sizes, and unfortunately we need explicit likelihoods in our empirical Bayes approach. In recent work [16], we have developed an efficient method for computing likelihoods under this model. The key idea is that the above dynamics can be written as

$$\mathbf{v}_{k+1} = \mathbf{M}_k^T \mathbf{v}_k$$

where $\mathbf{v}_k$ is a vector of dimension $2N + 1$ where entry i is the probability that there are i haploids

bioRxiv preprint doi: https://doi.org/10.1101/2023.05.19.541520; this version posted May 21, 2023. The copyright holder for this preprint (which was not certified by peer review) is the author/funder, who has granted bioRxiv a license to display the preprint in perpetuity. It is made available under aCC-BY 4.0 International license.

that have the LOF allele in generation k, and $\mathbf{M}_k$ is a matrix where row i is the the probability mass function of the Binomial distribution in Equation 8 given that the allele frequency in generation k is $i/2N_k$. This formulation makes clear that we can obtain the likelihood of observing a given frequency at present given some initial distribution by performing a series of matrix-vector multiplications. Naively this would be prohibitively slow as $\mathbf{M}_k$ can be as large as $10^7 \times 10^7$, but in [16] we show that $\mathbf{M}_k$ is approximately highly structured — it is both approximately extremely sparse and approximately extremely low rank. Combining these insights we can perform matrix-vector multiplication that is provably accurate while reducing the runtime for matrix-vector multiplication from $O(N_k^2)$ to $O(N_k)$. Similar insights can be used to speed up the computation of equilibria, which we discuss in detail in [16]. Furthermore, as discussed above, we actually want to compute likelihoods conditioned on non-fixation of the LOF allele, but that is as simple as setting the column of $\mathbf{M}_k$ corresponding to fixation to 0, and then renormalizing $\mathbf{v}$. We precompute these likelihoods for each possible pair of mutation rates (to and from the LOF allele) across a range of s_{het} values (100 log-linearly spaced points between 10^{-8} and 1, as well as 0). We describe how we set the mutation rates and the population sizes implicit in $\mathbf{M}_k$ below.

Modeling misannotation of LOFs

Under the likelihood described above, and as seen in Figure 2**A**, positions where a LOF variant could occur, but no LOF alleles are observed are slight evidence in favor of selection, while high frequency variants are extremely strong evidence against selection. Meanwhile, we suspect that many variants that are annotated as causing LOF actually have little to no effect on the gene product due to some form of misannotation. If these misannotated variants evolve effectively neutrally, they can reach high frequencies and cause us to artifactually infer artificially low levels of selection. These misannotated variants can be particularly problematic for approaches that combine frequencies across all LOFs within a gene to obtain an aggregate gene-level LOF frequency [1,2,4].

LOEUF [12] and pLI [11] avoid this problem by throwing away all frequency information except for whether an LOF is segregating or not. While this approach is more robust, the ignored frequency information is extremely useful for estimating the strength of selection. For example, consider a gene where we expect to see 5 unique LOFs under neutrality and we see 3 segregating LOFs. This might seem like weak or negligible constraint (O/E = 0.6), but if those 3 sites are all highly mutable and the variants at those sites are each only present in a single individual, then it is plausible that this gene is quite constrained.

To take full advantage of the information in the LOF frequencies while remaining robust to misannotation, we take a composite likelihood approach [91], closely related to the Poisson random field assumption commonly used in population genetics [89]. We approximate gene-level likelihoods as a product of variant level likelihoods

$$p^{(i)}\left(\boldsymbol{y}^{(i)} \mid s_{\text{het}}^{(i)}\right) \approx \prod_{j=1}^{J_i} p_{\text{variant}}\left(\boldsymbol{y}_j^{(i)} \mid s_{\text{het}}^{(i)}\right),$$

where $\boldsymbol{y}^{(i)}$ is a vector of the observed allele frequencies at each possible LOF site in gene i, and $s_{\text{het}}^{(i)}$ is the selection coefficient for having a heterozygous loss-of-function of gene i. Under this formulation, we can easily model misannotation by assuming that each LOF independently has

bioRxiv preprint doi: https://doi.org/10.1101/2023.05.19.541520; this version posted May 21, 2023. The copyright holder for this preprint (which was not certified by peer review) is the author/funder, who has granted bioRxiv a license to display the preprint in perpetuity. It is made available under aCC-BY 4.0 International license.

some probability of being misannotated, p_{miss}, and that misannotated variants evolve neutrally:

$$p_{\text{variant}}\left(\mathbf{y}_j^{(i)} \mid s_{\text{het}}^{(i)}\right) = (1 - p_{\text{miss}})p_{\text{DTWF}}\left(\mathbf{y}_j^{(i)} \mid s_{\text{het}}^{(i)}\right) + p_{\text{miss}}p_{\text{DTWF}}\left(\mathbf{y}_j^{(i)} \mid 0\right).$$

Using this formulation, we can take full advantage of the rich information included in the exact sample frequencies of each LOF variant, while still being robust to occasional misannotation. In practice, we precompute p_{variant} using a grid of p_{miss} values, and then to obtain the likelihood at arbitrary values of s_{het} and p_{miss} we linearly interpolate in log-likelihood space. Below, we discuss our approach for setting p_{miss}.

Given a probability of misannoation, we can then calculate a posterior probability that any given variant has been misannotated. We include a table of these misannotation probabilities for all possible LOFs in Supplementary Table XXX.

As an example of the importance of correcting for misannotation, we consider the case of the gene PPFIA3 (ENSG00000177380). This gene has a LOEUF score of 0.12 and so appears very constrained, but in an early version of our model where we did not incorporate variant misannotation, we inferred a posterior mean value of s_{het} of $\sim 2 \times 10^{-4}$, which is right at the border of being nearly neutral. Inspecting the LOF data for this gene, we find that all potential LOFs are either not observed or observed in a single individual, except for a single splice donor-disrupting variant at 16% frequency. There are no obvious signs indicating that this variant is misannotated (e.g., in terms of coverage or mappability). If we model misannotation, however, we find that this variant is likely misannotated (posterior probability of misannotation > 99.999%), and as a result we estimate extremely strong selection against gene loss-of-function (posterior mean s_{het} of $\sim$ 0.234). Indeed, a single autosomal dominant missense variant in this gene is suspected to have caused a number of severe symptoms including developmental delay, intellectual disability, seizures, and macrocephaly in an Undiagnosed Diseases Network participant (`https://undiagnosed.hms.harvard.edu/participants/participant-159/`) [92].

Modeling the X chromosome

We must slightly modify our model when applying it to the X chromosome. Because males only have one copy of the X chromosome, there are only 3/4 as many X chromosomes as autosomes (assuming an approximately equal sex ratio). As a result, when dealing with the X chromosome we scale all population sizes to 3/4 of the size used for the autosomes (rounded to the nearest integer). We also need to slightly modify the expected frequency in the next generation. We assume haploid selection in males with strength s_{hom}, and diploid selection in females with selection coefficients s_{het} and s_{hom} for individuals heterozygous and homozygous for the LOF variant respectively. This selection results in modified allele frequencies in the pool of males and females, and the we assume that each chromosome in the next generation has 1/3 probability of coming from a male, and 2/3 probability of coming from a female. This means that the expected frequency in the next generation is 1/3 times the post-selection frequency in males plus 2/3 times the post-selection frequency in females. Variants within the pseudoautosomal regions on the X are modeled identically to variants on the autosomes. Agarwal and colleagues also considered selection on the X in the context of LOF variants, with a model similar to that described here [4].

bioRxiv preprint doi: https://doi.org/10.1101/2023.05.19.541520; this version posted May 21, 2023. The copyright holder for this preprint (which was not certified by peer review) is the author/funder, who has granted bioRxiv a license to display the preprint in perpetuity. It is made available under aCC-BY 4.0 International license.

Model parameters

Our model has three key parameters — the mutation rate, the demographic model (i.e., population sizes through time), and the probability that different variants are misannotated.

We obtained mutation rates from gnomAD [12, Supplemental Dataset 10], which take into account trinucleotide context and methylation level (for CpG to TpG mutations). In our population genetics model, we assume that there are only two alleles (a functional allele and an LOF allele), whereas in reality there are four nucleotides. We approximate the rate of mutating from the functional allele to the LOF allele as being the sum of the mutation rates from the reference nucleotide to any nucleotide that might result in LOF. For example, if the reference allele is A, and either a C or a T would result in LOF, then we say that the rate at which the functional allele mutates to the LOF allele is the rate at which A mutates to C in this context plus the rate at which A mutates to T in this context. For the rate of back mutation from the LOF allele to the functional allele, we compute a weighted average of the rates of each possible LOF nucleotide back-mutating to any possible non-LOF nucleotide, weighed by the probability that the original non-LOF nucleotide mutated to that particular LOF nucleotide. Continuing our previous example, suppose A mutates to C at rate 1×10^{-8} and A mutates to T at a rate 1.5×10^{-8}. Then conditioned on there having been a single mutation resulting in a LOF variant, there is a $1/2.5 = 0.4$ chance that the LOF is C and 0.6 chance that the LOF is T. We then compute the back mutation rate as 0.4 times the rate at which C mutates to A in this context plus the rate at which C mutates to G in this context (since both A and G do not result in LOF) plus 0.6 times the rate at which T mutates to A in this context plus the rate at which T mutates to G in this context. Implicitly this scheme assumes that the flanking nucleotides in the trinucleotide context do not change, and we further assume that all mutations resulting in CpGs result in unmethylated CpGs.

For the population sizes in each generation, we used the “CEU” model inferred in [73] using the 1000 Genomes Project data [93]. This model was also used in [4]. Population sizes under this model are relatively constant before 5156 generations ago (approximately 155 thousand years ago) and the effects of strong selection are relatively insensitive to all but the most recent population sizes, so for a computational speedup we assumed that the population size was constant prior to 5156 generations ago. Recently, [4] found that this CEU model underestimates the number of low frequency variants and that changing the population size to 5,000,000 for the most recent 50 generations provides a better fit to the data. We used both demographic models and found qualitatively similar results, with slightly better fit provided by the modified model, so we used that demographic model for all subsequent analyses. In both cases, we modified the most ancient population sizes, which are relatively constant, to be actually constant to speed up likelihood calculations. The demographic models are presented in Supplementary Figure 1.

The only remaining model parameter is p_{miss} the probability that any given LOF is misannotated. Throughout we focus on LOFs that either introduce early stop codons, disrupt splice donors, or disrupts splice acceptors. Given that predicting which variants have these different consequences involves different bioinformatic challenges, we inferred separate misannoatation probabilities p^c_{miss} for $c \in \{\text{stop codon}, \text{splice donor}, \text{splice acceptor}\}$. Below we write p_{miss} for the collection of these three misannotation parameters. To get a rough estimate of these parameters and avoid excessive computational burden, we took an h-likelihood approach [94,95]. That is, we jointly maximized the likelihood across all genes with respect to their selective constraints as well

bioRxiv preprint doi: https://doi.org/10.1101/2023.05.19.541520; this version posted May 21, 2023. The copyright holder for this preprint (which was not certified by peer review) is the author/funder, who has granted bioRxiv a license to display the preprint in perpetuity. It is made available under aCC-BY 4.0 International license.

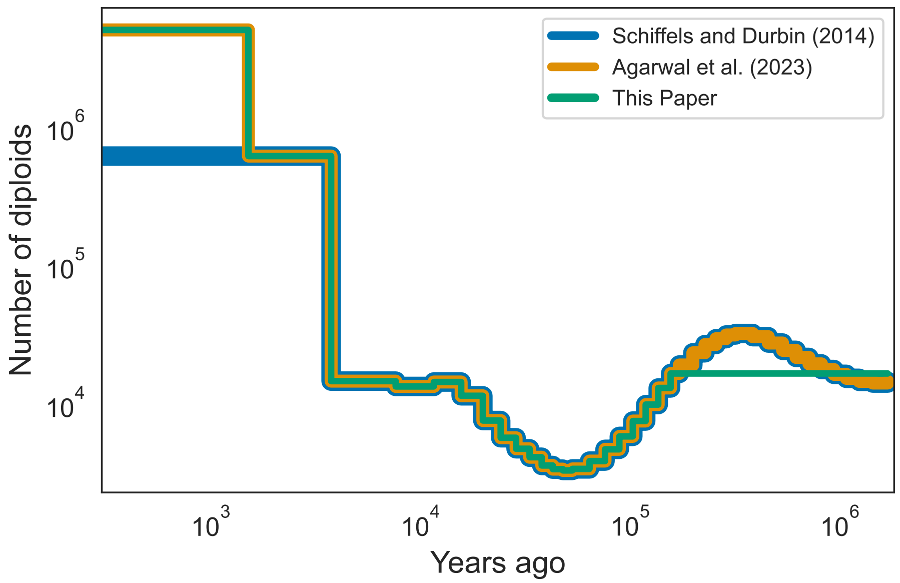

Supplementary Figure 1: CEU Demography inferred by Schiffels and Durbin [73], modified by Agarwal and colleagues [4], and further modified for this paper.

as the the three misannotation probabilities that are shared across all genes:

$$\max_{p_{\text{miss}}, s_{\text{het}}^{(1)}, \ldots, s_{\text{het}}^{(M)}} \sum_{i=1}^{M} \log p\left(\mathbf{y}^{(i)} \mid s_{\text{het}}^{(i)}, p_{\text{miss}}\right).$$

This approach of just using the maximum likelihood estimates of s_{het} for each gene contrasts with the standard empirical Bayes approach, which would involve marginalizing out the unknown s_{het} values. Yet, this marginalization step depends on the prior on s_{het}, which we learn via our `NGBoost` framework. As a result, we would need to repeatedly run our `NGBoost` framework as an inner loop to perform the standard empirical Bayes approach on p_{miss}. For our application, these values are nuisance parameters, and the results are relatively insensitive to their exact values so we opted for this simpler h-likelihood approach. Ultimately, we estimate that the probability of misannotation is 0.7%, 6.1%, and 8.4% for stop codons, splice donors, and splice acceptors respectively.

bioRxiv preprint doi: https://doi.org/10.1101/2023.05.19.541520; this version posted May 21, 2023. The copyright holder for this preprint (which was not certified by peer review) is the author/funder, who has granted bioRxiv a license to display the preprint in perpetuity. It is made available under aCC-BY 4.0 International license.

C Feature processing and selection

We compiled 10 types of gene features from several sources:

1. *Gene structure.* Gene structure features were derived from GENCODE gene annotations (Release 39) [76]. Such features include the number of transcripts and, for the primary transcript of each gene (the transcript tagged `Ensembl_canonical`), the number of exons as well as the length and GC content of the transcript, total coding region, 5′ UTR, and 3′ UTR.

2. *Gene expression.* We used gene features from 77 bulk and single-cell RNA-seq datasets, processed and derived in [96]. These datasets can be grouped into 24 categories representing tissues, cell types, and developmental stage (Table 6). For each dataset, features were derived separately from all data and from individual cell clusters (for example, gene loadings on principal components). In addition, features were derived from comparisons between clusters (for example, t-statistics for differential expression). Finally, we include a metric, τ, that summarizes the tissue-specificity of gene expression [97].

3. *Biological pathways and Gene Ontology terms.* First, we included previously curated biological pathway features [96, 98]. In addition, to include GO terms that capture additional known relationships between genes, we downloaded Biological Pathway (BP), Molecular Function (MF), and Cellular Component (CC) terms [99] with at least 10 member genes using the procedure described in [10]. Features for each gene were encoded as binary indicators of the gene's membership in the pathways and GO terms.

4. *Connectedness in protein-protein interaction (PPI) networks.* We included previously computed measures of the connectedness of protein products of genes in PPI networks [10]. Connectedness was calculated as the number of interactions per protein weighted by the interaction confidence scores.

5. *Co-expression.* First, we included previously computed measures of the connectedness of genes in co-expression networks [10], where connectedness measures the relative number of neighbors of each gene in the network, averaged over tissues. Next, for each gene, we derived features representing its co-expression with other genes (i.e. correlation in their expression levels across samples). To do this, we downloaded from the GeneFriends database a co-expression network derived from GTEx RNA-seq samples [100,101], calculated the variance in the co-expression for each gene, and kept the 6,000 most variable genes. Then, we included the co-expression with each of these 6,000 genes as a feature.

6. *Gene regulatory landscape.* Gene regulatory features include the counts and properties of the enhancers and promoters that regulate each gene. First, we included the number of promoters per gene estimated by the FANTOM consortium using Cap Analysis of Gene Expression [10, 102]. Next, for each gene, we calculated the number, summed length, and summed score of enhancer-to-gene links predicted using the Activity-By-Contact (ABC) approach [49,103], where an enhancer is considered linked to a gene if its ABC score is ≥ 0.015. We computed separate features for each of 131 biosamples. We also included features derived by aggregating over all biosamples for both ABC enhancers and predicted enhancers

bioRxiv preprint doi: https://doi.org/10.1101/2023.05.19.541520; this version posted May 21, 2023. The copyright holder for this preprint (which was not certified by peer review) is the author/funder, who has granted bioRxiv a license to display the preprint in perpetuity. It is made available under aCC-BY 4.0 International license.

from the Roadmap Epigenomics Consortium [10, 104, 105]—these feature include the number of biosamples with an active enhancer element, the total number of enhancer elements, the total number of enhancer elements after taking merging enhancer domains, the total length of the merged domains, and the average total enhancer length in an active cell type. Finally, we included the enhancer-domain score for each gene [9] as a feature.

7. *Conservation across species.* For each gene, we calculated the mean and 95th percentile phastCons scores over the gene's exons for multiple alignments of 7, 17, 20, 30, and 100 vertebrate species to the human genome [106]. We downloaded phastCons Scores from `https://hgdownload.soe.ucsc.edu/goldenPath/hg38/`. In addition, we included the fraction of coding sequence (CDS) or exons constrained across 240 mammals or 43 primates sequenced in the Zoonomia project [107], with constraint determined by the per-base phyloP [108] or phastCons score. Zoonomia data were downloaded from `https://figshare.com/articles/dataset/geneMatrix/13335548`.

8. *Protein embedding features.* We included as features the embeddings learned by an autoencoder (ProtT5) trained on protein sequences [109]. Embeddings were downloaded from `https://zenodo.org/record/5047020`. The embedding for each protein is a fixed-size vector that captures some of the protein's biophysical and functional properties. For each gene with more than one protein product, we averaged the embeddings of the proteins for that gene.

9. *Subcellular localization.* We included as features the subcellular localization of each protein and whether the protein is membrane-bound or soluble, as predicted by deep neural networks trained on the ProtT5 protein embeddings [109, 110]. Possible subcellular classes included nucleus, cytoplasm, extracellular space, mitochondrion, cell membrane, endoplasmatic reticulum, plastid, Golgi apparatus, lysosome or vacuole, and peroxisome. Predictions were one-hot encoded, and for each gene with more than one protein product, we summed the predictions for the gene's proteins. Predictions were downloaded from `https://zenodo.org/record/5047020`.

10. *Missense constraint.* We included a measure of each gene's average intolerance to missense variants (UNEECON-G score) [111]. UNEECON-G scores incorporate variant-level features to account for differences in the effects of missense variants on gene function.

In addition to these 10 groups of features, we included a binary indicator for whether the gene is located on the X chromosome. Genes in the pseudoautosomal regions were categorized as autosomal.

After compiling these features (total of 65,383), we performed feature selection to minimize the practical complexity of training on such a large feature set and the complexity of the resulting model. First, we removed features with zero variance and features where the Spearman correlation of the feature values with O/E (the ratio of observed over expected unique LOF variants, computed using gnomAD data) was less than 0.1 or had a nominal p-value $\geq$ 0.05. Next, we performed simultaneous feature selection and an initial round of hyperparameter tuning using the `shap-hypetune` package, which uses Bayesian optimization to identify a set of features and hyperparameters that minimize the loss of a machine learning model fit on the training data. Specifically, we fit gradient-boosted trees using `XGBoost` to predict O/E from the gene features; we chose to

bioRxiv preprint doi: https://doi.org/10.1101/2023.05.19.541520; this version posted May 21, 2023. The copyright holder for this preprint (which was not certified by peer review) is the author/funder, who has granted bioRxiv a license to display the preprint in perpetuity. It is made available under aCC-BY 4.0 International license.

perform feature selection using `XGBoost` rather than `NGBoost` as training `XGBoost` models is substantially faster, and because we expect features/hyperparameters that perform well for `XGBoost` to also perform well for `NGBoost`. For each set of hyperparameters, `shap-hypetune` performs backward step-wise selection by removing the k least influential features (we chose $k = 1000$ and calculated influence using SHAP scores) at each step. Finally, we performed further feature selection using `shap-hypetune` by fixing the hyperparameters and performing backward step-wise selection with $k = 50$. Ultimately, we included 1,248 features in the model.

bioRxiv preprint doi: https://doi.org/10.1101/2023.05.19.541520; this version posted May 21, 2023. The copyright holder for this preprint (which was not certified by peer review) is the author/funder, who has granted bioRxiv a license to display the preprint in perpetuity. It is made available under aCC-BY 4.0 International license.

D Estimating additional gene properties using GeneBayes

GeneBayes is a flexible framework that can be used to infer other gene-level properties of interest beyond s_{het}. In Figure 6, we presented a schematic of the key components of GeneBayes that users should specify, which we describe in more detail now.

First, users should specify the gene features to use as predictors. We expect the gene features we use for s_{het} estimation to work well for other applications, but GeneBayes supports any choice of features. In particular, GeneBayes can handle categorical and continuous features without feature scaling, as well as features with missing values.

Next, users should specify the form of the prior distribution. GeneBayes supports the distributions defined by the `distributions` package of `PyTorch`. GeneBayes also supports custom distributions, as long as they implement the methods used by GeneBayes (i.e. `log_prob` and `sample`) and are differentiable within the `PyTorch` framework.

Finally, users need to specify a likelihood function that relates their gene property of interest to observed data. The likelihood can be specified in terms of a `PyTorch` distribution, or as a custom function.

After model training, GeneBayes outputs a per-gene posterior mean and 95% credible interval for the property of interest. For each parameter in the prior, GeneBayes also outputs a metric for each feature that represents the contribution of the feature to predictions of the parameter.

In the next section, we describe in more detail the two example applications that we outlined in Figure 6.

Example applications

Differential expression

In this example, users have estimates of log-fold changes in gene expression between conditions and their standard errors from a differential expression workflow, and would like to estimate log-fold changes with greater power (e.g. for lowly-expressed genes with noisy estimates).

Likelihood We define $\ell_{\text{DE}}^{(i)}$ and ℓ_i as the estimated and true log-fold change in expression respectively for gene i, and s_i as the standard error for the estimate. Then, we define the likelihood for ℓ_i as

$$\ell_{\text{DE}}^{(i)} \mid \ell_i \sim \text{Normal}(\ell_i, s_i^2).$$

Prior We describe two potential priors that one may choose to try. The first is a normal prior with parameters μ_i and σ_i:

$$\ell_i \sim \text{Normal}(\mu_i, \sigma_i^2).$$

The second is a spike-and-slab prior with parameters π_i, μ_i, and σ_i, which assumes that gene i

bioRxiv preprint doi: https://doi.org/10.1101/2023.05.19.541520; this version posted May 21, 2023. The copyright holder for this preprint (which was not certified by peer review) is the author/funder, who has granted bioRxiv a license to display the preprint in perpetuity. It is made available under aCC-BY 4.0 International license.

only has a π_i probability of being differentially expressed:

$$z_i \sim \text{Bernoulli}(\pi_i)$$
$$\ell_i | z_i \sim \begin{cases} 0, & \text{if } z_i = 0 \\ \text{Normal}(\mu_i, \sigma_i^2), & \text{if } z_i = 1 \end{cases}$$

Variant burden tests

In this example, users have sequencing data from patients with a disease or (if calling *de novo* mutations) sequencing data from family trios, and would like to identify genes with excess mutational burden in patients (e.g. an excess of missense or LOF variants). One approach is to infer the relative risk for each gene (denoted as γ_i for gene i), defined as the expected ratio of the number of variants in patients to the number of variants in healthy individuals.

Likelihood Let E_i be the number of variants we expect to observe for gene i given the study sample size and sequence-dependent mutation rates (e.g. expected counts obtained using the mutational model developed by [82]). Next, let O_i be the number of variants observed in patients for gene i. Then, we define the likelihood for η_i as

$$O_i \mid \eta_i \sim \text{Poisson}(\eta_i E_i).$$

Prior Because η_i is non-negative, one may want to choose a gamma prior with parameters α_i and β_i:

$$\eta_i \sim \text{Gamma}(\alpha_i, \beta_i).$$

bioRxiv preprint doi: https://doi.org/10.1101/2023.05.19.541520; this version posted May 21, 2023. The copyright holder for this preprint (which was not certified by peer review) is the author/funder, who has granted bioRxiv a license to display the preprint in perpetuity. It is made available under aCC-BY 4.0 International license.

Gene	s_{het}	LOEUF
RPL11	0.75	0.3
RPL18	0.72	0.28
RPL5	0.71	0.17
RPL35A	0.67	0.41
RPL15	0.61	0.27
RPL26	0.61	0.38
RPS15A	0.61	0.56
RPS7	0.60	0.31
RPS10	0.60	0.27
RPS26	0.58	0.48
RPL27	0.56	0.48
RPS24	0.48	0.59
RPS29	0.40	1.2
RPS27	0.31	0.64
RPS28	0.26	0.8
RPL35	0.25	0.72

Supplementary Table 1: **LOEUF and s_{het} for ribosomal proteins**

bioRxiv preprint doi: https://doi.org/10.1101/2023.05.19.541520; this version posted May 21, 2023. The copyright holder for this preprint (which was not certified by peer review) is the author/funder, who has granted bioRxiv a license to display the preprint in perpetuity. It is made available under aCC-BY 4.0 International license.

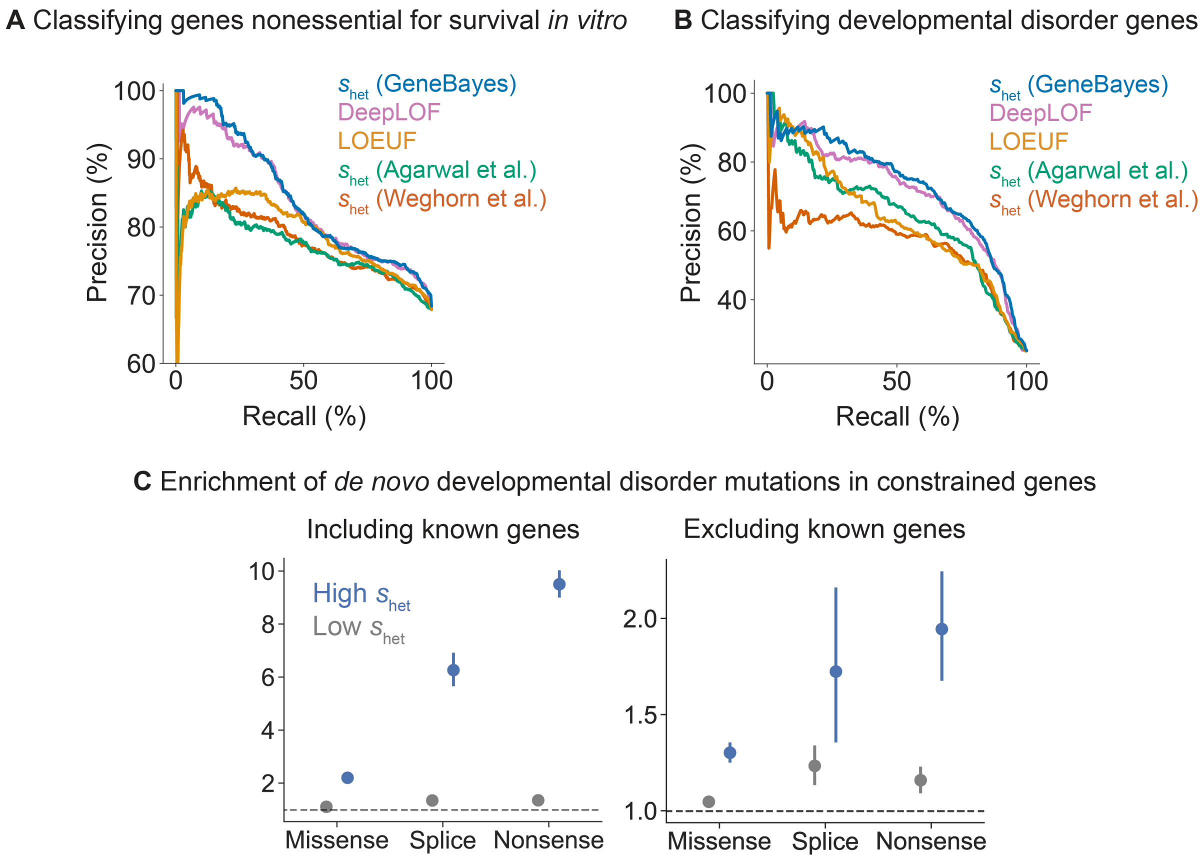

Supplementary Figure 2: **Additional validation analyses.** **A)** *Precision-recall curves comparing the performance of* s_{het} *estimates from GeneBayes against other constraint metrics in classifying non-essential genes.* **B)** *Precision-recall curves comparing the performance of* s_{het} *against other constraint metrics in classifying developmental disorder genes.* **C)** *Enrichment of de novo mutations in patients with developmental disorders, calculated as the observed number of mutations over the expected number under a null mutational model. We plot the enrichment of missense, splice, and nonsense variants in the 10% of genes considered most constrained by* s_{het} *(blue) and in all other genes (gray), including (left) and excluding (right) known developmental disorder genes. Bars represent 95% confidence intervals.*
